# An Atypical Way of Restoring an Internally Mangled Implant With the Use of a Cast Post: A Case Report

**DOI:** 10.7759/cureus.69632

**Published:** 2024-09-18

**Authors:** Kaustubh Thakare, Nishita Jaju, Priyanka Jaiswal, Aishwarya Rathod, Kshipra Kawadkar

**Affiliations:** 1 Department of Periodontics, Vidarbha Youth Welfare Society Dental College and Hospital, Amravati, IND; 2 Department of Periodontics, Sharad Pawar Dental College and Hospital, Datta Meghe Institute of Higher Education and Research, Wardha, IND

**Keywords:** complications, custom post and core, implant abutment, screw loosening, screw retrieval

## Abstract

Implant-supported prostheses can lead to biological or mechanical failures. The loosening of the abutment screw and its fracture are the most common technical complications. Recovery of fractured parts is quite challenging due to the unavailability of a universal standard screw retrieval kit on the market. Various techniques have been described earlier for retrieval of fractured fragments or internal thread damage, but with no optimum success and eventually leading to implant failure and removal, which causes additional surgical exposure and economic impediment. Hence, this clinical report explains the viable option for such a situation using an alternative technique and simple armamentarium available in regular clinic setups.

## Introduction

Nowadays, dental implants are the most popular and predictable method of replacing missing tooth/teeth and completely edentulous patients. Implants have the highest success rate of 97% to 99% when proper case selection along with acceptable treatment protocol, appropriate placement, and ideal prosthetic design are provided [1-3]. Despite success and predictability in the long term, complications and failure in implants happen in some cases [4,5]. Nevertheless, complications also have been reported [4]. These failures in implant treatment can be broadly categorized into biological and mechanical [6]. The surface treatments of dental implants have increased the predictability of osseointegration, thereby reducing the prevalence of biological complications. Mechanical complications are more frequently occurring and they include problems related to components of the superstructure which include the loosening of screws and their fracturing [7]. The complication rates studied for five years for single-tooth implant prostheses due to screw loosening consist of around 8.8%, that of prosthesis dislodgement constitutes 4.1%, and fracture of prosthetic component is 3.5% [8]. Fatigue from biomechanical overload, active fit of prosthesis, or a few errors occurring during the manufacturing of implant components can lead to fracture of the implant [9,10]. Less abutment-related complications are seen with internal types of implants than with external ones [11]. Retrieval of the abutment screw above the position of the fixture can be easily done with the help of a hemostat or tweezers [12]. When the screw cannot be removed conservatively in cases of abutment failure under the fixture, due to expensive kits available for screw retrieval and customized for that particular implant system, regularly used dental instruments like rotary can be used to retrieve the fractured screw. However, the problem associated with the use of such instruments is that the internal threads of the screwhole can be damaged, and the implant may be of no use. Because of this, implantologists often prefer to either retrieve and remove the implant and place a new one, or submerge the implant and leave it covered with soft tissue. The present report gives a detailed description of a procedure for saving an implant with damaged internal threads by a cast post and fabrication of a new crown from the original parts.

## Case presentation

**Figure 1:**
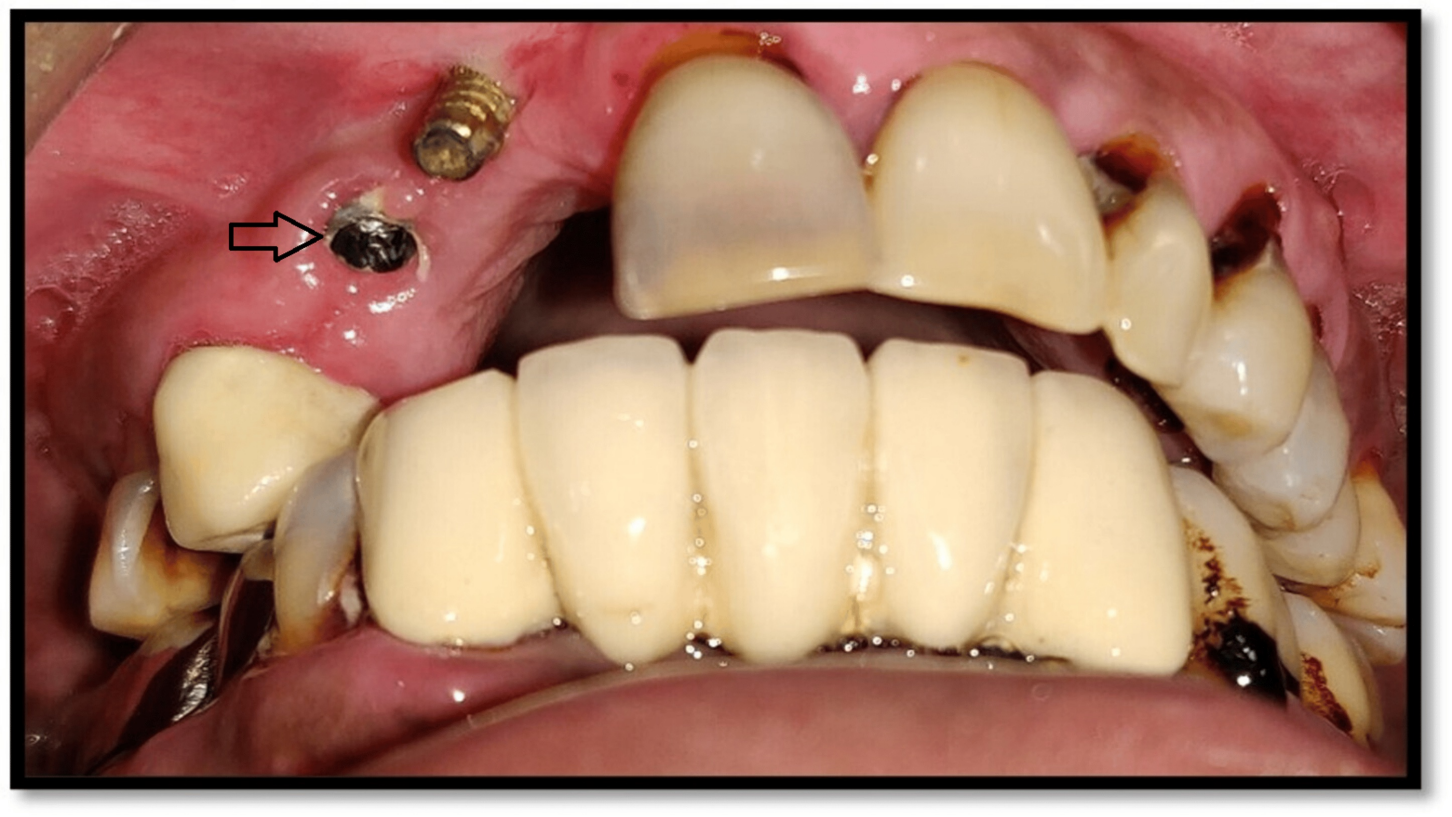
Clinical view showing fractured abutment screw

A 45-year-old male patient had a chief complaint of loosening of her implant prosthesis at the left upper middle tooth region due to which he visited a private dental clinic. According to the dental history, the implant had been placed five years before by some other implantologist. A screw-retained porcelain prosthesis was placed for masticatory function on the implant with the patient's consent. After removing the implant prosthesis, it was observed that the screw of the implant abutment was fractured (Figure 1).

**Figure 2:**
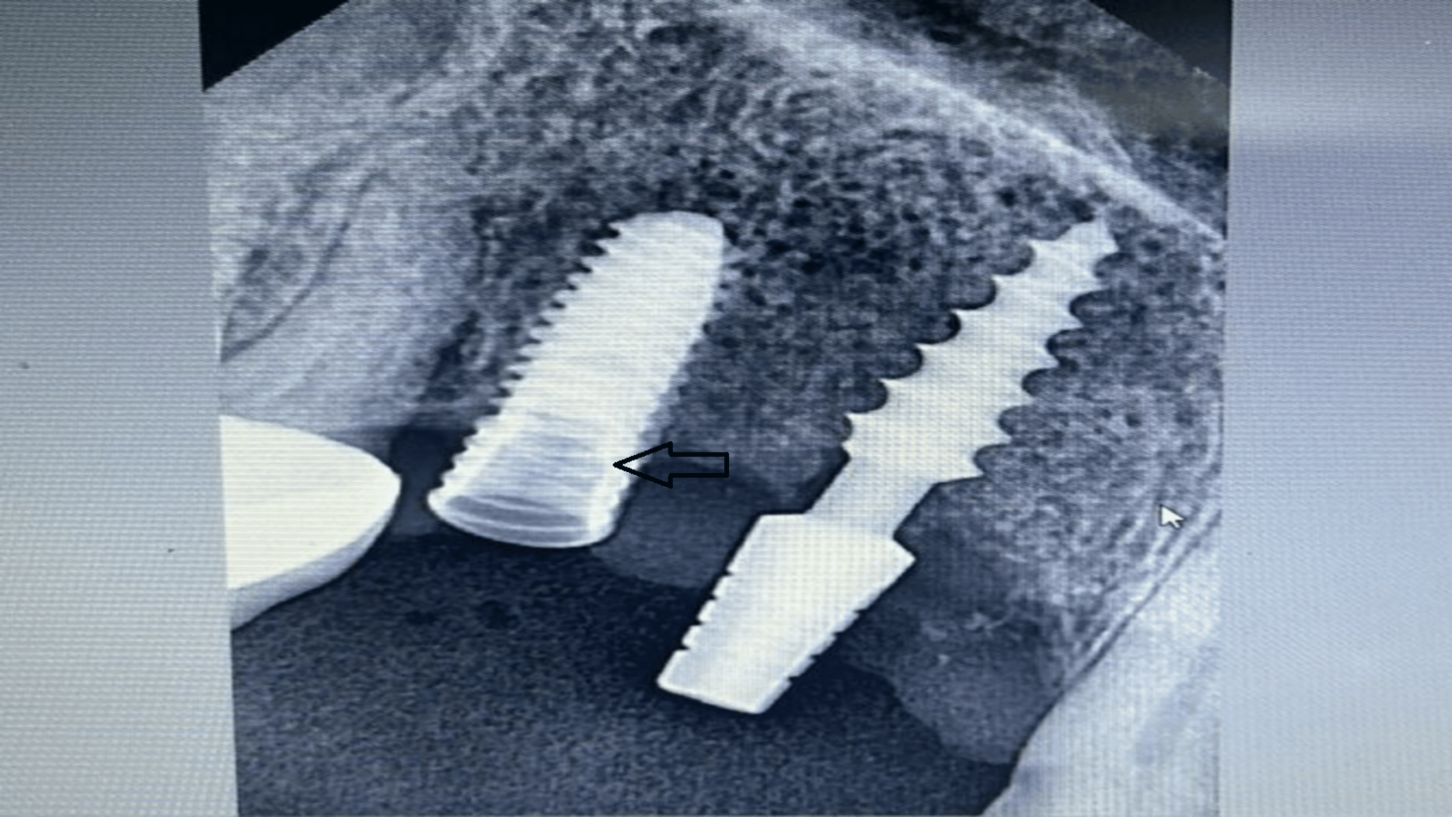
Radiographic view showing fractured abutment screw

**Figure 3:**
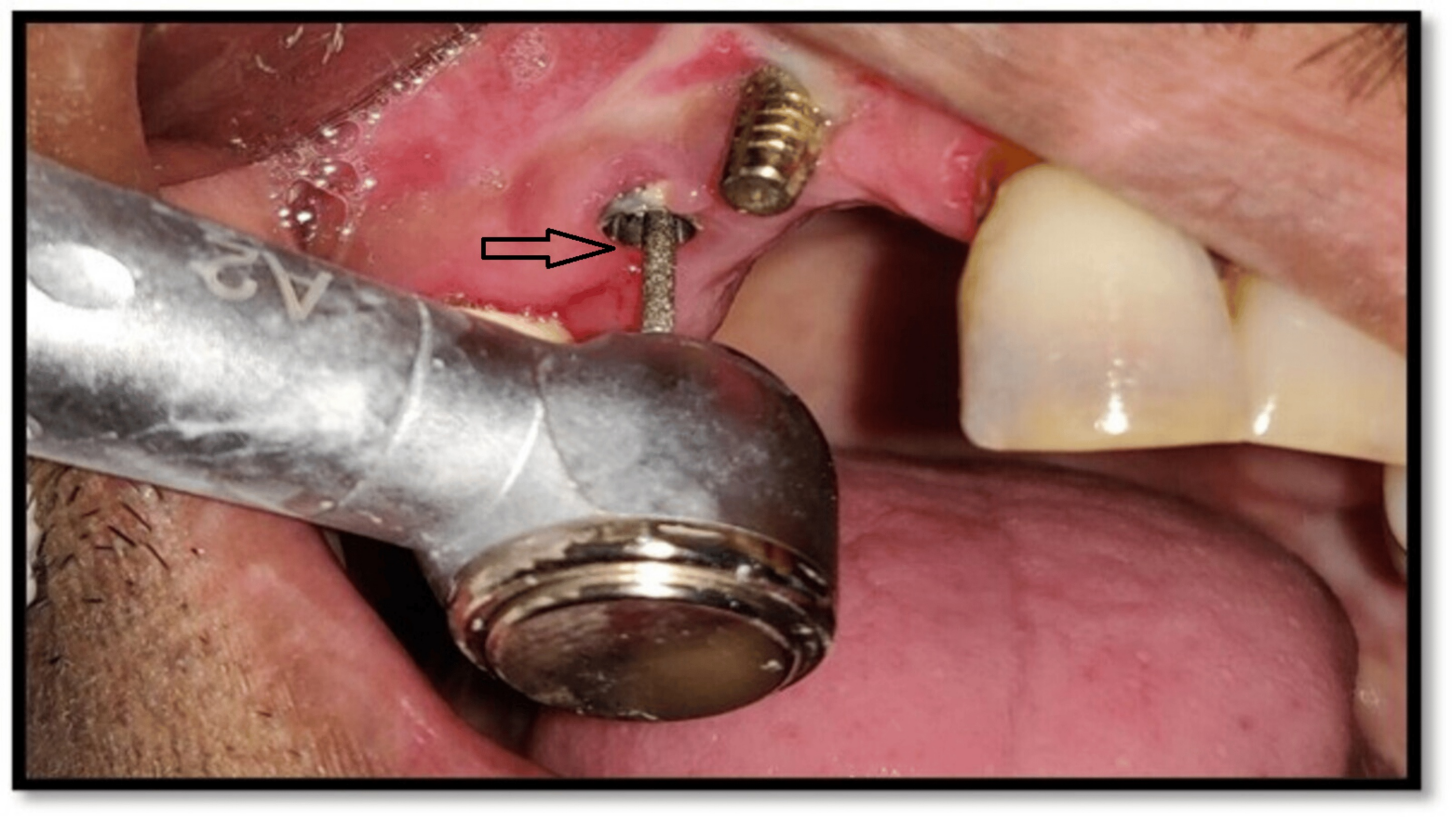
Use of carbide burs while trying to retrieve the fractured screw

Radiographic examination revealed no significant marginal bone loss around implant sides, ruling out the chances of peri-implantitis. No cardinal signs of inflammation were visible on clinical examination. The implant was well osseointegrated. The fracture screws deep from the soft tissue were observed in the periapical view of the radiovisiograph (RVG) (Figure 2) and were seen 3 mm deep from the soft tissue. Different techniques, such as the use of common dental equipment like that of an explorer and ultrasonic scaler, were tried for screw removal with no success. The screw removal was quite challenging as it was located at the third screw thread, and additionally, the left-over screw had preloaded torque. Thus, a high-speed motorized handpiece with a carbide bur was used for the careful removal of the fragments of the leftover screw retaining inside the implant (Figure 3).

While in the process of removing the screw fragments, the internal threads were damaged, causing the failure of the healing abutment to completely connect to the implant. In the given scenario, removal of the implant and replacing it with a newer one would have been considered the easiest option. However, the patient refused implant removal and could not afford the finances for a new implant placement. Furthermore, the radiograph revealed good osseointegration of the implant. So, it was a prudent choice to save the implant. Thus, removal of the existing implant was not considered a choice of treatment.

**Figure 4:**
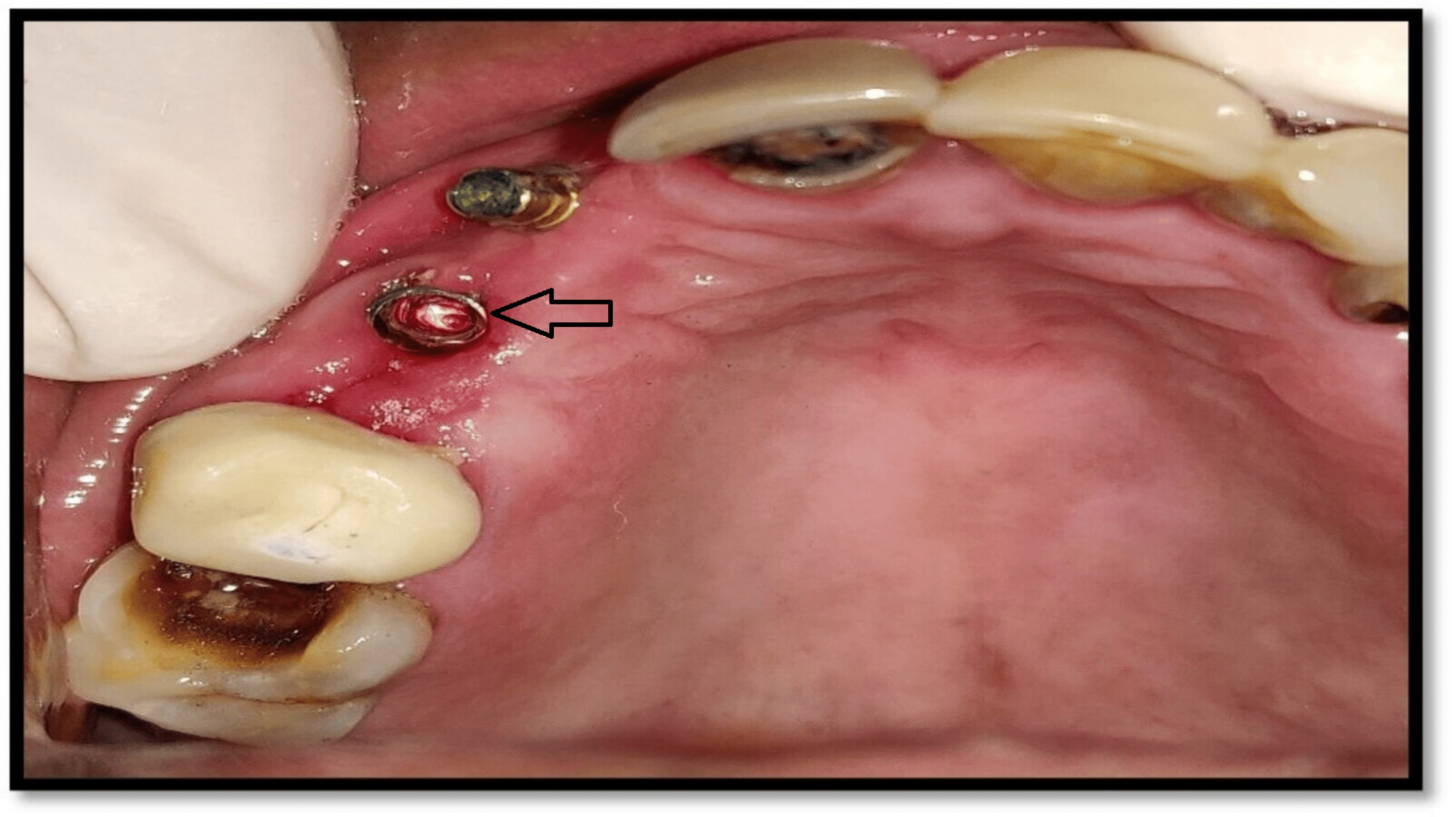
Completely smoothened inner surface of implant

First, we considered the basic idea of practice by submerging the pre-existing implant and later fabricating a fixed partial three-unit prosthesis. But, then considering the healthy and sound existing adjacent bone and implants, the treatment plan of fabricating a cast post-restoration was planned on the existing implant. Prior to recording the cast postimpression, the overgrown gingival tissue was excised with the due action of local anesthesia for a clearer clinical view. Sequentially, the inner surface of the implant was completely smoothened using a carbide bur used for metal cutting and a fine-polished round taper diamond bur in a high-speed handpiece under copious irrigation (Figure 4).

**Figure 5:**
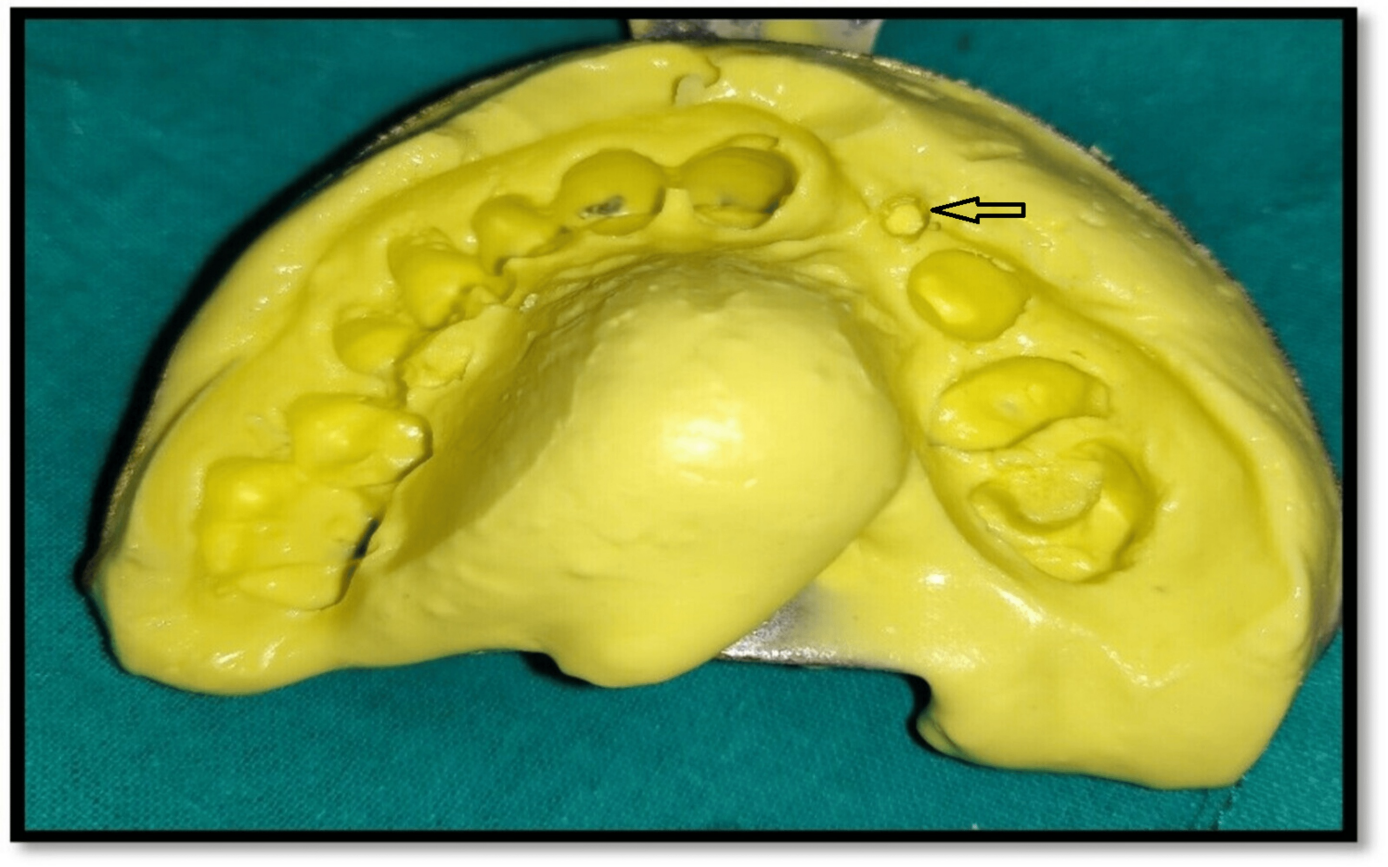
Impressions made for customization of cast post

To start with, the impression was recorded with polyvinylsiloxane (PVS) material (Figure 5).

**Figure 6:**
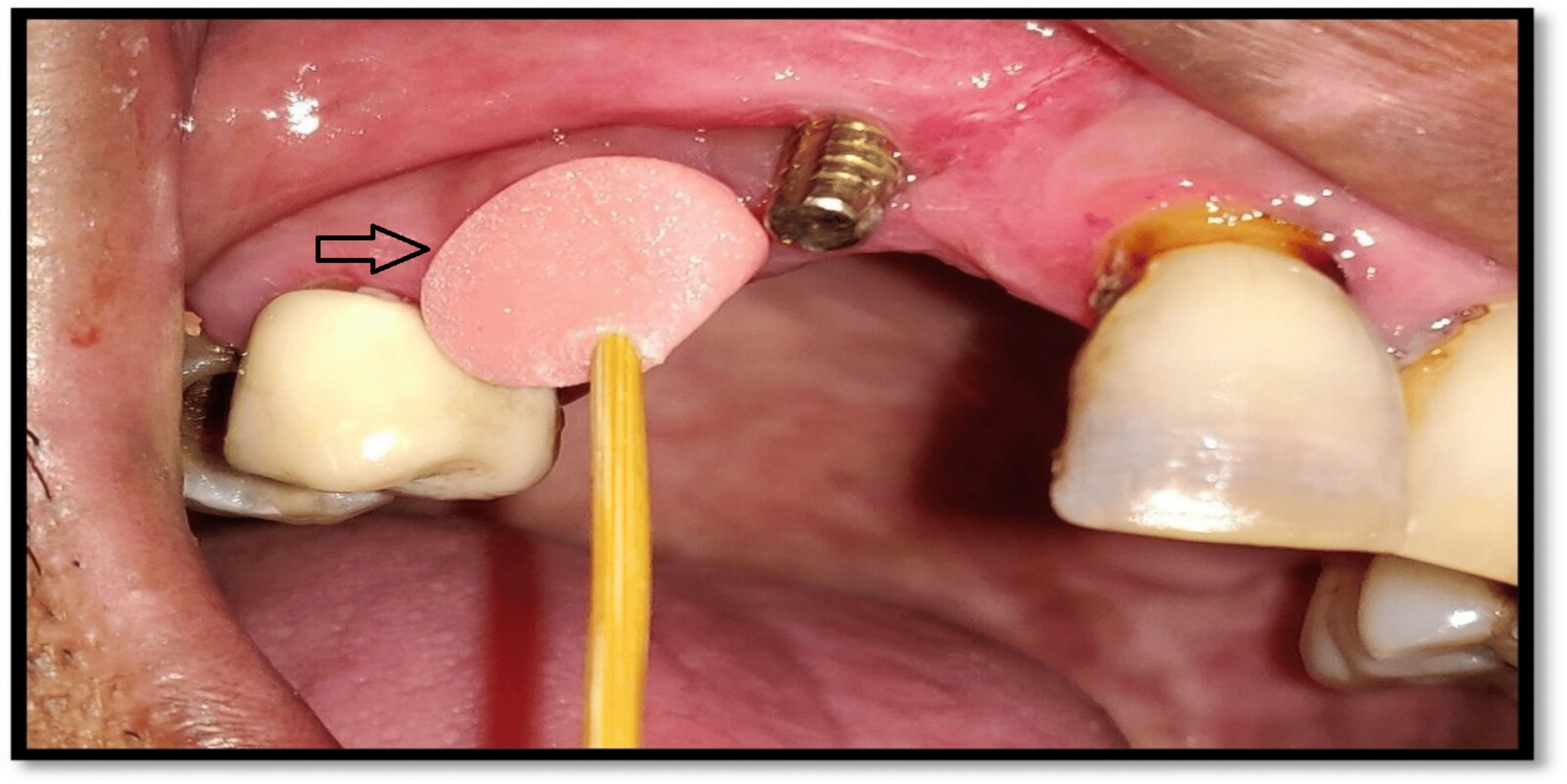
Fabrication of customized cast post on to the implant using pattern resin

After recording impressions, the inner aspect of the fixture was restored temporarily with a temporary restoration material. A temporary restorative material was used instead of a gingival former to prevent the overgrowth of soft tissue around the fixture platform. A master cast was fabricated with regular dental stone. The cast post was designed using a wax pattern and later invested (Figure 6).

**Figure 7:**
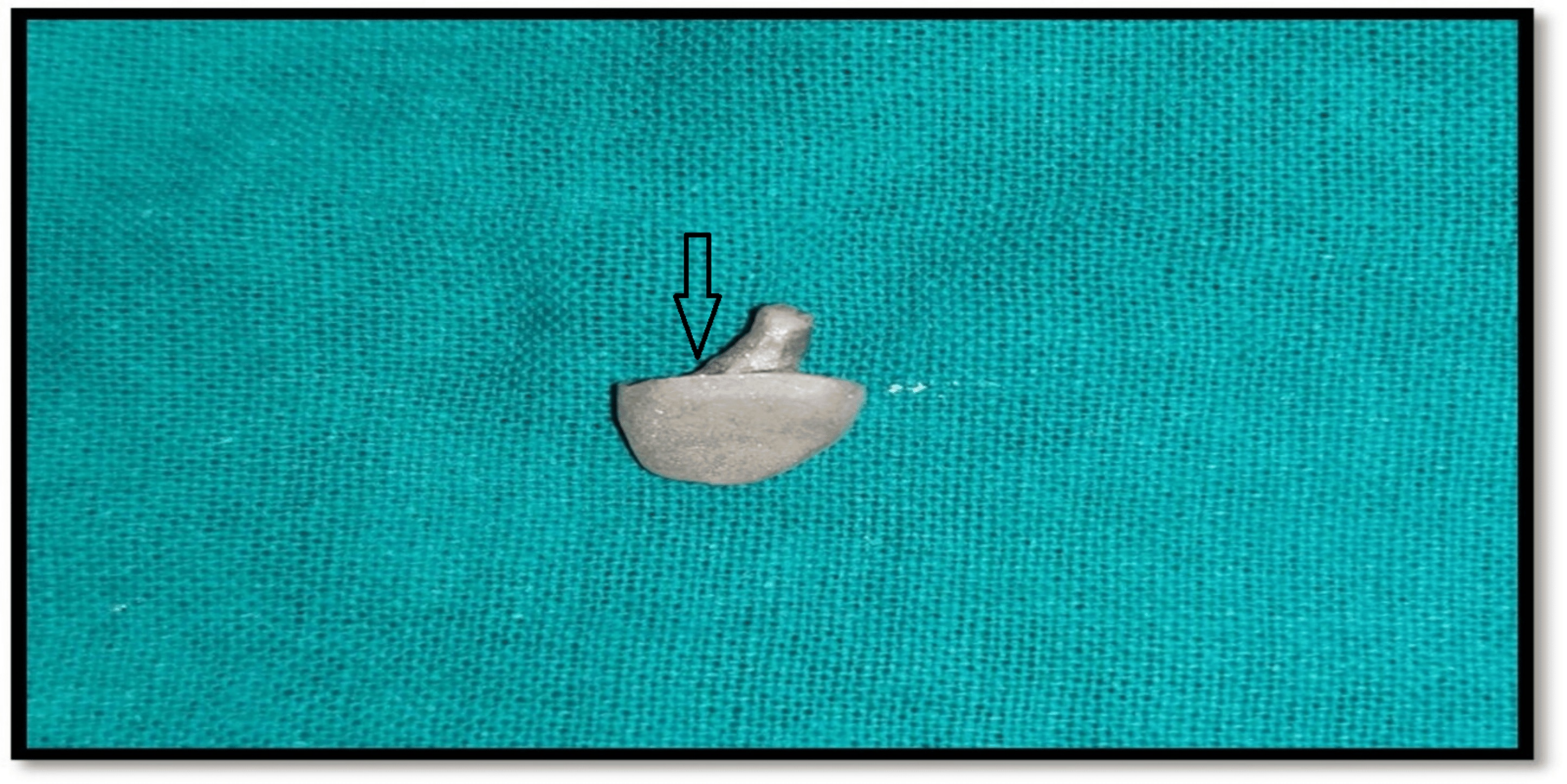
After casting of core build up for placing on to the abutment

Subsequently, it was burned out and cast using a nickel-chromium alloy (Figure 7).

**Figure 8:**
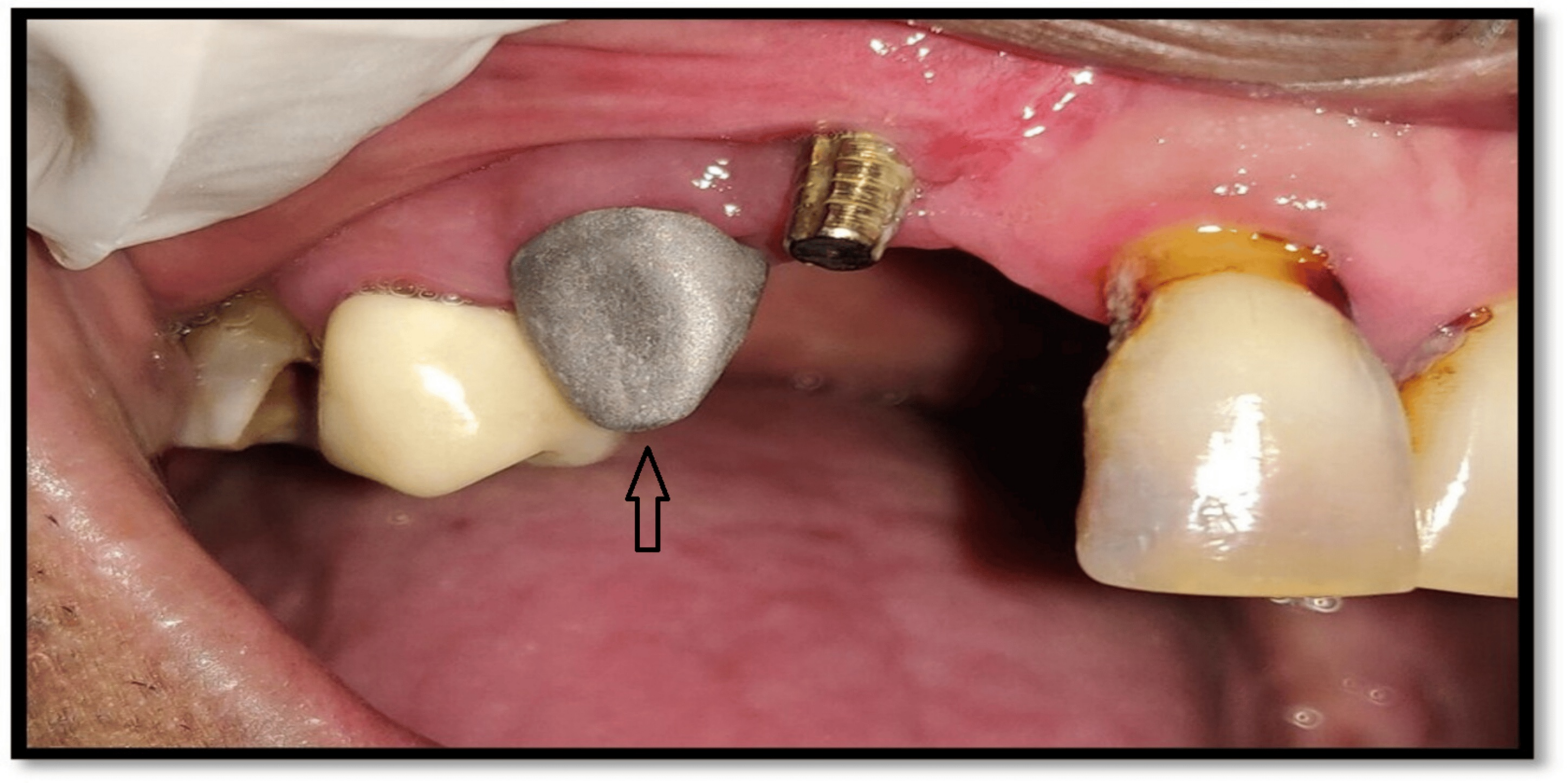
Trial of customized core build up done

On a later date, the metal try-in for checking internal and marginal fits was carried out (Figure 8).

**Figure 9:**
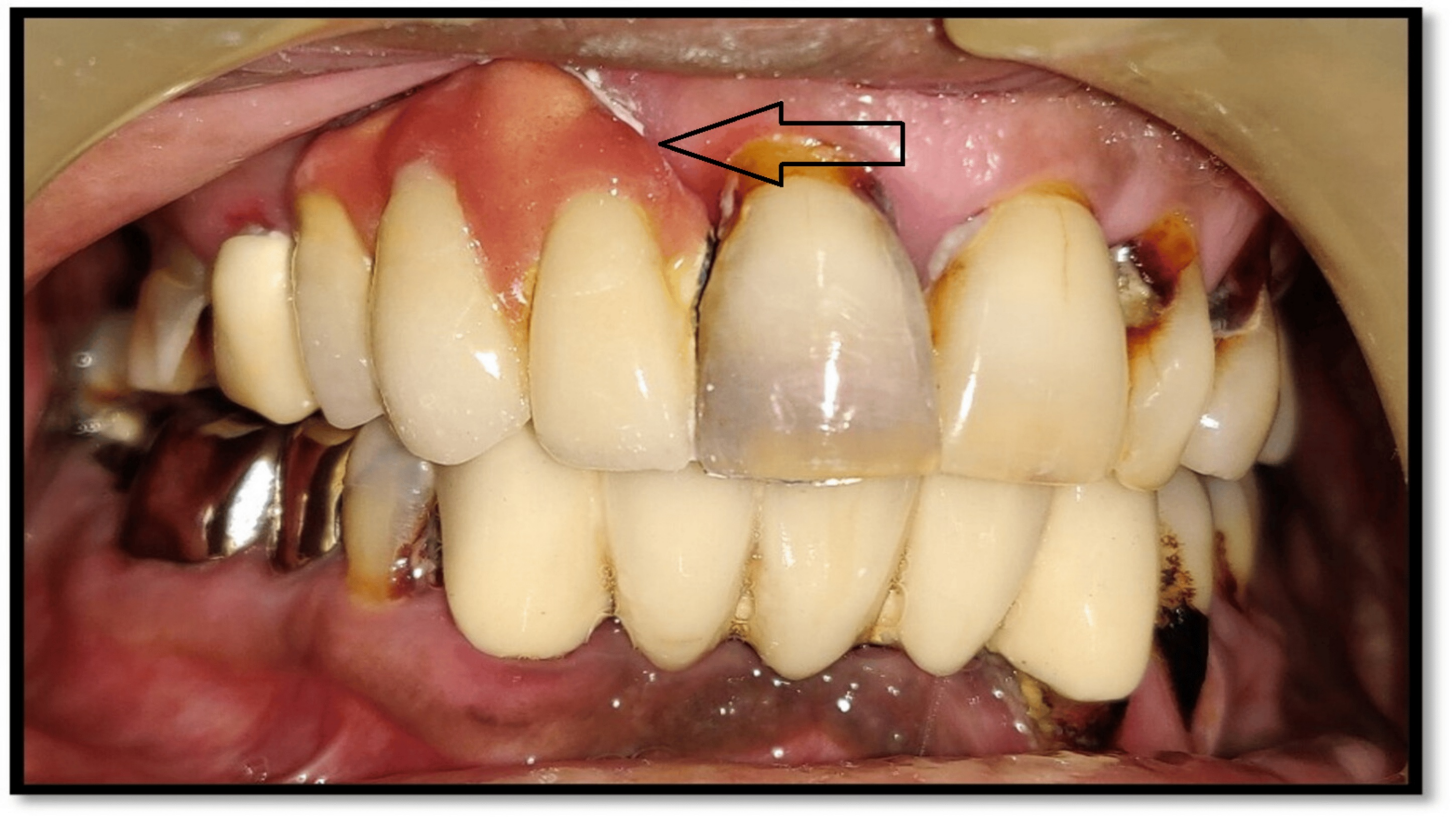
Final cement retained prosthesis delivered to the patient

Adequate space was left for the ceramic firing above the metal. The passive fitting of the cast post was verified into the fixture, setting a finish line margin that was equi-gingival. The prosthesis of the upper arch was fabricated of a metal ceramic. The patient had a trial of a newly casted prosthesis and oral evaluation and adjustment were performed. Following the principles for implant-supported prostheses, occlusal adjustments were done. The occlusion after the prosthesis was meticulously evaluated for high points to avoid paramount force, which could potentially cause implant failure. After a few corrections, the fixture from the inner side was completely dried, and bonding of the cast post to the fixture using luting cement was done (Figure 9).

Then, excessive cement was removed and radiographs were taken post-implant prosthesis; the patient was given instructions to avoid hard and sticky food. Regular follow-up for proper occlusion was scheduled after every three months. One year postoperatively, no complications, neither with the implant nor with the prosthesis, were seen.

## Discussion

The main cause leading to mechanical complications is screw loosening and subsequently screw fracturing. Here, educating the patient plays an important role since most implant screw loosening can abruptly lead to screw fracture. After any mobility at the peri-implant area is noticed, it is advisable to report to the dental clinic in no time to avoid screw fracturing. Although abutment screw fracture seldom occurs in clinical practice, dealing with such situations can be a piece of work for clinicians [13-15]. If the fracture lies coronal to the head of the implant, it is quite easier to remove it with the help of an artery or hemostat. However, if the fracture lies apical to the head, then removal should be such that it should not damage the implant fixture’s inner surface, which can complicate the situation. If the thread at the inner surface is damaged during removal, the abutment screw cannot be simply replaced. It is known in our general practice to remove the entire implant when the internal threads are damaged. However, the procedure for removal of the existing failed implant and placement of a new implant is time-consuming and a tedious procedure and may be costly too. Moreover, in the process, excessive alveolar bone reduction might be inevitable so as to remove the fixture. The success rate of these replanted implants is comparatively lesser than that of the initial implant [16]. In such a helpless situation, fabricating a cast with post and core-supported restoration can save the osseointegrated fixture [17]. Thus, probing is a boon to implant dentistry. In this case, the patient left the loosened screw unattended for a long time and did not report to the clinic after the mobility of the prosthesis, which resulted in a screw fracture due to masticatory forces. In this case, we tried our best to fabricate a customized cast post and cement prosthesis for an internally damaged implant without trying to retrieve the implant. A similar procedure has never been used in any of the studies done previously, that too with a one-year follow-up. Additionally, the advantage of this procedure was the short treatment period compared to new implant placement, the lack of second surgery, and that it could be afforded by economically weakened patients as well. Hence, this can be considered a newer and simpler technique for restoring good osseointegrated implants with abutment screw fractures.

## Conclusions

Utilization of customized cast posts may be done as an abutment for implants damaged internally in cases of osseointegrated implants. It is an uncomplicated and effective treatment for restoring internally mangled implants without the need for replacement.
